# Is Lymphocyte C-Reactive Protein Ratio Useful for Predicting Survival in Patients with Non-Metastatic Soft Tissue Sarcoma?

**DOI:** 10.3390/cancers14215214

**Published:** 2022-10-24

**Authors:** Tomoki Nakamura, Tomohito Hagi, Kunihiro Asanuma, Akihiro Sudo

**Affiliations:** Department of Orthopaedic Surgery, Mie University Graduate School of Medicine, 2-174 Edobashi, Tsu 514-8507, Japan

**Keywords:** lymphocyte- to- C-reactive protein ratio, soft tissue sarcoma, survival

## Abstract

**Simple Summary:**

We hypothesized that lymphocyte to C-reactive protein ratio (LCR) could predict survival in 132 patients with soft tissue sarcoma (STS). This study aimed to determine whether LCR before treatment can predict disease-specific survival (DSS) and event-free survival (EFS). The 5-year DSS in patients with higher and lower LCR was 86.5% and 52.8%, respectively (*p* < 0.001). Patients with lower LCR had worse survival than those with higher LCR. The 5-year EFS in patients with higher and lower LCR was 66.2% and 31.2%, respectively (*p* < 0.001). On Receiver operating characteristic analysis, however, there was no significant difference in the area under curve (AUC) between CRP level (AUC = 0.72) and LCR (AUC = 0.711). LCR was found to be a poor prognostic factor for oncological outcomes using multivariate analysis. although ROC analysis could not show the superiority of LCR to CRP for predicting oncological outcomes in patients with STS.

**Abstract:**

Background: Recently, the lymphocyte-to-CRP ratio (LCR) was found to have a prognostic role in many cancers. However, the clinical significance of LCR in patients with soft tissue sarcoma (STS) has not yet been established. This study aimed to determine whether LCR can predict disease-specific survival (DSS) and event-free survival (EFS) in patients with STS. Methods: In this study, 132 patients were enrolled. The mean follow-up periods were 76.5 months. Blood examinations were performed prior to treatment for all patients. Results: The 5-year DSS in patients with higher and lower LCR was 86.5% and 52.8%, respectively (*p* < 0.001). Patients with lower LCR had worse survival than those with higher LCR. The 5-year EFS in patients with higher and lower LCR was 66.2% and 31.2%, respectively (*p* < 0.001). On Receiver operating characteristic analysis, however, there was no significant difference in the area under curve (AUC) between CRP level (AUC = 0.72) and LCR (AUC = 0.711). Conclusions: LCR may be a prognostic factor for predicting oncological events in multivariate analysis, although ROC analysis could not show the superiority of LCR to CRP for predicting oncological outcomes in patients with STS.

## 1. Introduction

Soft tissue sarcoma (STS) is a rare heterogeneous tumor and accounts for 1–2% of all malignancies in adults [1]. Even after radical treatment of primary STS, as many as 50% of these patients experience local recurrence and distant metastasis [2,3]. Therefore, easy, well-known, and low-cost markers may help identifying a high risk for tumor relapse. Inflammation plays a critical role in the development, progression, clinical presentation, and diagnosis of tumors. Elevated C-reactive protein (CRP) has been shown to be a useful predictor for predicting poor survival in patients with STS [4,5,6]. In all type of peripheral blood cells, Lymphocytes are the most important type, which works against cytotoxic responses to cancer cells. Lymphocytes also work against proliferation of cancer cells, migration of cancer cells, and invasion of cancer cells [7]. Therefore, in the case of lymphocytopenia, the cellular immune system cannot work properly, and inflammatory reaction cannot be appropriately established. Several studies have shown that absolute lymphocyte counts (ALC) decrease in tumor-induced inflammation and that there is a relationship between the decrease in ALC and poor prognosis [8,9]. Recently, the lymphocyte-to-CRP ratio (LCR) was related to the prognosis in many solid cancers such as hepatocellular carcinoma, colorectal cancer, and esophageal cancer [10,11,12,13]. However, the role of LCR in patients with STS has not yet been established. In this study, we aimed to clarify the role of LCR in predicting disease-specific survival (DSS) and event-free survival (EFS) in adult patients with non-metastatic STS. Further aims were to compare the prognostic values of LCR, CRP levels, and lymphocyte count.

## 2. Materials and Methods

A total of 132 patients with STS who were surgically treated between January 2002 and December 2018 were reviewed. Patients with local recurrence, distant metastasis at initial presentation were excluded from this study. Patients who were referred for additional resection after unplanned excision were also excluded. Finally, Patients with insufficient blood examination data were excluded. The median and mean follow-up periods were 59.5 and 76.5 months, respectively (range: 3.6–207). All patients were examined by chest CT images for finding possible distant metastasis and deciding pretreatment staging. Pathological diagnosis and tumor grades were determined for all patients without myxoid liposarcomas by pathologists using the French Federation of Cancer Centers Sarcoma Group (FNCLCC) grading system. Pathological diagnosis and tumor grades were determined for patients with myxoid liposarcomas using World Health Organization criteria. Blood examinations were performed before any treatment for all patients.

The study was performed in accordance with the ethical standards of the Clinical Research Ethics Review Committee of the Mie University Hospital (H2020-224). Written informed consent was waived, and an “opt-out” option was permitted wherein the patients had an opportunity to deny participation in this study.

### Statistical Analysis

The statistical associations were evaluated between clinical backgrounds and LCR using the Mann–Whitney U-test and Kruskal–Wallis test for continuous variables and the chi-squared test for categorical variables. Correlations between LCR, CRP, and ALC were analyzed using Spearman’s rank correlation analysis. The DSS was defined as the time from initial surgery to the date of sarcoma-related death, or the date when the patient was documented to be alive. EFS was defined as the time from surgery were taken from the date of the initial treatment for the primary tumor to the date when the patient was documented to be alive without any oncological events or the date with local recurrence or metastasis. The DSS and EFS were estimated using the Kaplan–Meier method. Cumulative survival was compared using the log-rank test. Univariate and multivariate analyses were performed using the Cox proportional hazards model. The statistical significant variables identified in the univariate analysis included in multivariate analysis A *p* value < 0.05 was regarded as showing statistical significance. Receiver operating characteristic (ROC) analysis was performed to determine the threshold of LCR, CRP, and ALC at risk of relapse or death within 3 years after surgery. All statistical analyses were performed with the EZR graphical user interface (Saitama Medical Centre, Jichi Medical University, Saitama, Japan) for R (The R Foundation for Statistical Computing, Vienna, Austria). This package is a modified version of R Commander frequently used in biostatistics to add statistical functions.

## 3. Results

### 3.1. Patient, Tumor, and Treatment Characteristics

Characteristics of patients are presented in Table 1. The mean and median age of the patients at diagnosis were 63 and 66 years (range: 21–89), respectively. The patients comprised 79 males and 53 females. The mean sizes at diagnosis were 8.9 cm, with a range of 2 to 30 cm. One hundred and twenty patients had high-grade (grade 2; 38 patients, grade 3; 82 patients), and 12 had low-grade (grade 1) tumors.

Histologically, 29 tumors were undifferentiated pleomorphic sarcomas, 28 were myxofibrosarcomas, 22 were leiomyosarcomas, 13 were de-differentiated liposarcomas, 11 were myxoid liposarcomas, 8 were malignant peripheral nerve sheath tumors, 6 were synovial sarcomas, 5 were fibrosarcomas, and 10 were other STSs.

The STS arose 55 at the thigh, 17 at the leg, 11 at the buttock, 10 at the upper arm, 9 at the chest wall, 8 at the back, and 22 at other sites. We included 3 retroperitoneal STSs. The tumor depth was as follows: superficial in 25 patients and deep-seated in 107 patients. Ten patients underwent adjuvant radiotherapy, and 31 patients underwent perioperative chemotherapy. 

### 3.2. The Relationship between the LCR and Clinical Characteristics

The median CRP level was 0.155 mg/dL (range: 0.01–20.4). The median ALC was 1770/µL (range: 380–3710). The median LCR was 13,518 (range: 19.9–273000). On ROC analysis, a value of 9608.696 was an appropriate threshold for LCR to identify if patients were at risk of death within 3 years (area under the curve [AUC] = 0.711; 95% confidence interval [CI]: 0.62–0.802). The relationship between the clinicopathological features and LCR is shown in Table 2. Tumor grade and size were significantly associated with LCR.

### 3.3. Overall DSS and Predictors of Mortality

**Figure 1:**
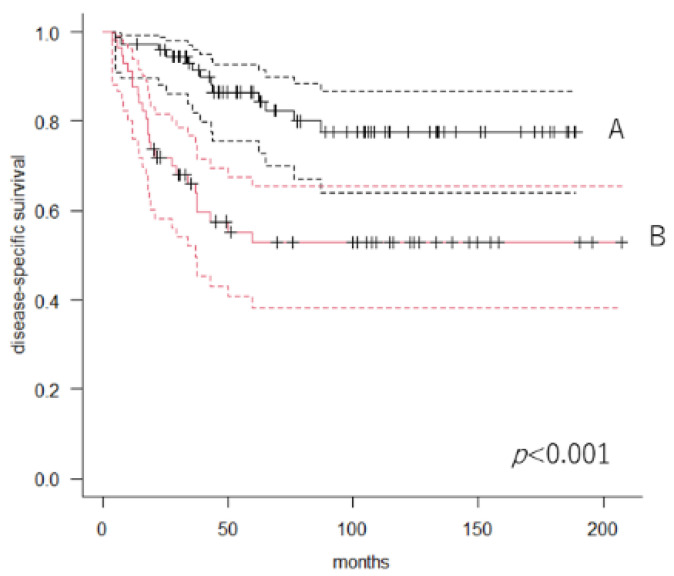
Kaplan–Meier curves showing disease-specific survival. A: Patients with a higher lymphocyte-to-C-reactive protein ratio. B: Patients with a lower lymphocyte-to-C-reactive protein ratio.

At the final follow-up, 99 patients (94/132; 71.2%) were alive, 38 had died of disease, and 8 had died of other disease. The 5-year DSS was 71.8% (95% CI: 62.8–79.1) in all 132 patients. Next, we divided the patients into two groups according to the value of LCR using ROC analysis. Patients with lower LCR had worse survival than those with higher LCR. The 5-year DSS in patients with higher (n = 75) and lower (n = 57) LCR was 86.5% (95% CI: 75.6–92.8) and 52.8% (95% CI: 38.3–65.4), respectively (*p* < 0.001, log-rank test) (Figure 1). 

A Cox proportional univariate analysis confirmed that tumor size (*p* < 0.001), CRP level (*p* < 0.001), and LCR (*p* < 0.001) (Table 3) were significant prognostic factors. Tumor size and CRP level were also prognostic factors in multivariate analysis.

### 3.4. Event-Free Rate and Predictors of Events

**Figure 2:**
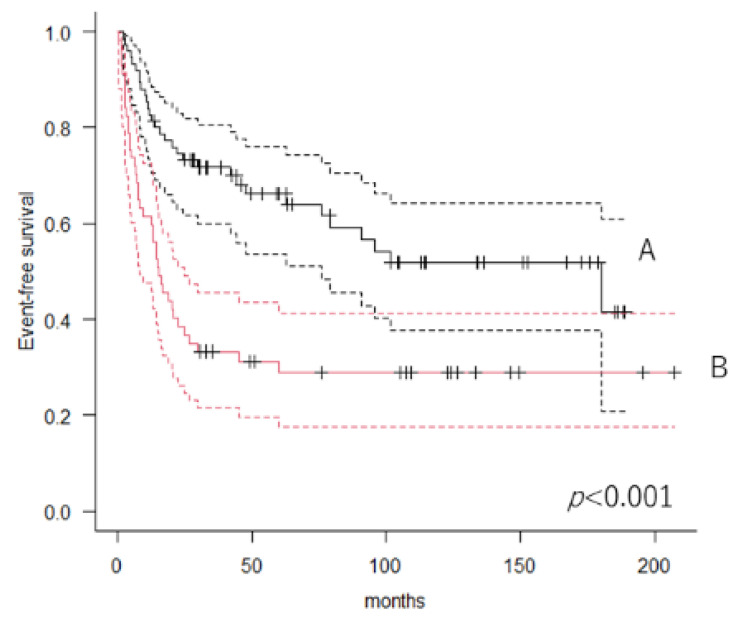
Kaplan–Meier curves showing event-free survival. A: Patients with a higher lymphocyte-to-C-reactive protein ratio; B: Patients with a lower lymphocyte-to-C-reactive protein ratio.

Of the 132 patients, 33 (25%) and 57 (43.1%) showed local tumor recurrence and distant metastasis, respectively. The 5-year event-free rate was 51% (95% CI: 41.9–59.3). Patients with lower LCR had worse survival than those with higher LCR. The 5-year EFS in patients with higher (n = 75) and lower (n = 57) LCR was 66.2% (95% CI: 53.7–76.1) and 31.2% (95% CI: 19.7–43.5), respectively (*p* < 0.001, log-rank test) (Figure 2). Cox proportional univariate analysis revealed that CRP level (*p* *<* 0.001), LCR (*p* < 0.001), tumor size (*p* < 0.001), age (*p* = 0.037), tumor depth (*p* = 0.038), and histological tumors (grade 3; *p* = 0.012) were significant predictors of the occurrence of events in STS patients. Multivariate analysis showed that CRP level (*p* < 0.001) and tumor size (*p* < 0.001) remained independent predictors of oncological events (Table 4). 

### 3.5. ROC Analysis

**Figure 3:**
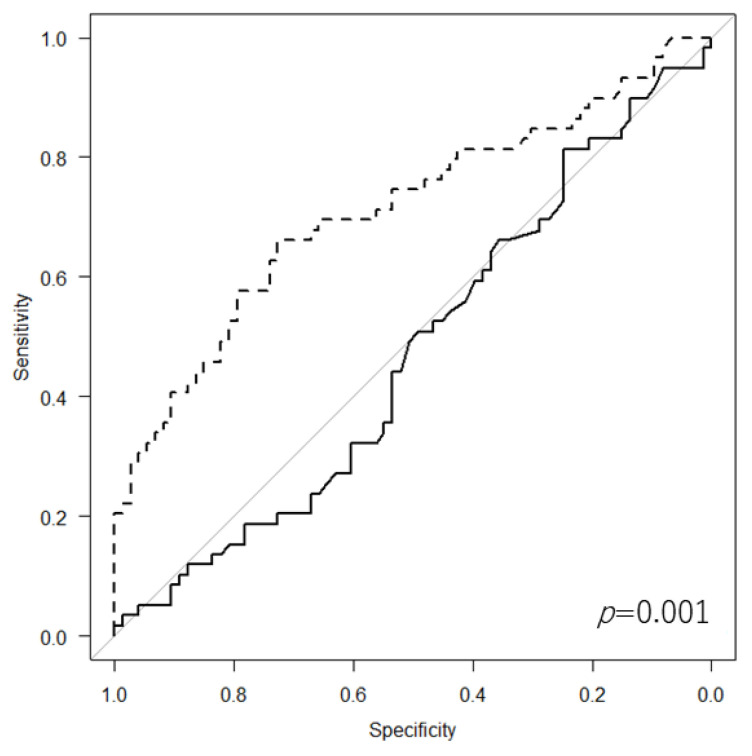
Receiver operating characteristic curve showing significant difference in area under the curve between absolute lymphocyte count (solid line) and lymphocyte-to-C-reactive protein ratio (dotted line).

**Figure 4:**
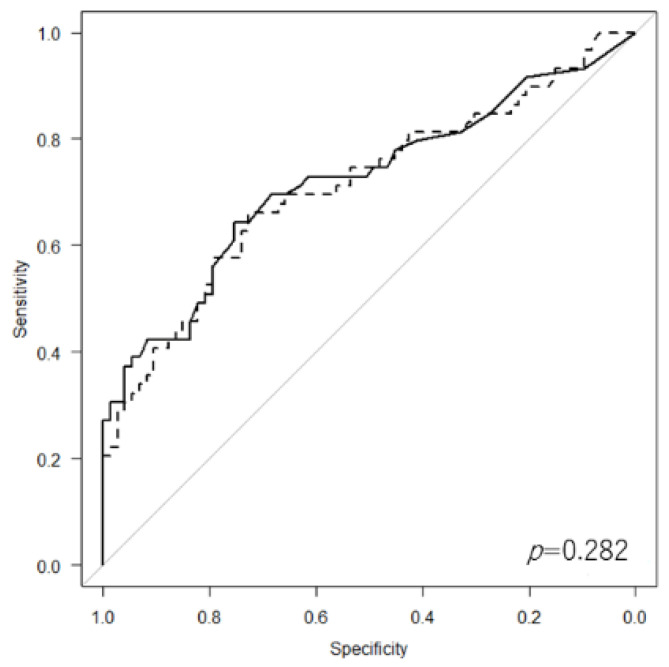
Receiver operating characteristic curve showing no significant difference in area under the curve between C-reactive protein level (solid line) and lymphocyte-to-C-reactive protein ratio (dotted line).

On ROC analysis, LCR was found to be a significantly appropriate marker (AUC = 0.711) for identifying whether patients were at risk of further diseases or death within 3 years compared with ALC (AUC = 0.471, *p* = 0.001) (Figure 3). However, there was no significant difference in the AUC between CRP level (AUC = 0.72) and LCR (*p* = 0.282) (Figure 4). 

## 4. Discussion

In the present study, we showed that LCR was associated with DSS and EFS in 132 patients with STS in univariate and multivariate analysis. However, on ROC analysis, the AUC of CRP for predicting survival was similar to that of LCR. We believe that the present results were affected by ALC.

We could not determine whether ALC was related to clinical outcomes, and ROC analysis showed that ALC was a poor indicator for predicting survival and oncological events. ALC has been associated with mortality in several cancers, including gastric, cervical, renal cell and lung cancers [7,8,14,15,16,17,18]. Brewster et al. reported that ALC at diagnosis were significantly inversely associated with overall survival in 634 patients with musculoskeletal sarcomas [19]. The median ALC was 1120/µL as per their study. They classified patients were classified as having lymphopenia if they had an ALC value less than 1000/µL. However, in the present study, we included only four patients with ALC values less than 1000/µL. Furthermore, the median ALC was 1770/µL. Teck et al. reported that the neutrophil-to-lymphocyte and lymphocyte-monocyte ratios were related to survival in 142 sarcoma patients, although they could not determine whether ALC was related to survival [20]. The median ALC was 1810/µL in their study. Therefore, we believe that the distribution of ALC may differ between cohorts; thus, the relationship between ALC and survival may be different. Nevertheless, the association of lymphocytes with host immunity and tumor progression should be considered. Lymphocyte plays prominent role in the tumor related immunity, which contribute to host defense [21,22,23,24]. Therefore, lymphocyte related prognostic tools, such as neutrophil to lymphocyte ratio and platelet to lymphocyte ratio, were reported in patients with STS [25,26,27,28].

In the present study, CRP was an independent prognostic marker for predicting survival and oncological events in multivariate analysis. CRP has been widely reported to be an independent prognostic marker in patients with STS [4,5,6,29,30]. The cohort with elevated CRP may be suitable for future clinical trials of intensive therapy.

This study had certain limitations. The retrospective nature of the study is a limitation. Although all patients had undergone chest CT scans and routine blood examinations to rule out the presence of metastasis and/or other cancers, other chronic disease that may increase CRP levels were not taken into consideration due to the lack of information. Furthermore, the distribution of ALC may differ between cohorts, even if the measurement was obtained before treatment in all patients. We also should consider the possible affection of small number of the patients with adjuvant radiotherapy and chemotherapy for oncological outcome in the present study, although adjuvant radiotherapy is not often performed if the adequate surgical margin is acquired in Japan. Further multicenter studies including other countries should be necessary to validate the present findings. 

## 5. Conclusions

LCR was found to be a poor prognostic factor for oncological outcomes in patients with STS using multivariate analysis, although ROC analysis could not show the superiority of LCR to CRP for predicting oncological outcomes in patients with STS.

## Figures and Tables

**Table 1:** Patient characteristics.

Variables	Unit	Value
Age	mean	63 years
	range	21–89 years
Sex	male	79
	female	53
Size	mean	8.9 cm
	range	2–30 cm
depth	superficial	25
	Deep	107
Grade	1	12
	2	38
	3	82
CRP	mg/dL	0.155
	Range	0.01–20.4
ALC	/µL	1770
	Range	380–3710

CRP: C-reactive protein, ALC: absolute lymphocyte count.

**Table 2:** Relationship between lymphocyte-to-C-reactive protein ratio and clinical characteristics.

Variables	Unit	Low LCR	High LCR	*p* Value
Age	mean	63	63	0.843
Sex	male	39	40	0.107
	female	18	35	
Size	mean	5.7	4.9	0.011
depth	superficial	7	18	0.117
	deep	50	57	
Grade	1	1	11	<0.001
	2	11	27	
	3	45	37	

LCR: lymphocyte-to-C-reactive protein ratio.

**Table 3:** Univariate and multivariate disease-specific survival analyses.

		Univariate Analysis			Multivariate Analysis		
Variables	Unit	HR	95% CI	*p* Value	HR	95% CI	*p* Value
Age	years	1.019	0.994–1.044	0.139			
Sex	female	1					
	male	0.8772	0.461–1.671	0.69			
Size	cm	1.116	1.063–1.171	<0.001	1.096	1.039–1.157	<0.001
Depth	deep	1					
	s.c	0.739	0.308–1.771	0.498			
Grade	1	1					
	2	3.18	0.403–25.1	0.273			
	3	5.372	0.731–39.53	0.099			
Histology	MFS	1					
	UPS	2.034	0.680–6.080	0.204			
	Others	1.874	0.715–4.915	0.202			
Cx	No	1					
	Yes	1.58	0.797–3.133	0.190			
LCR	high	1			1		
	Low	3.206	1.638–6.277	<0.001	1.836	0.868–3.883	0.112
CRP	mg/dL	1.177	1.109–1.25	<0.001	1.128	1.052–1.209	<0.001
ALC	/µL	0.999	0.999–1	0.35			

LCR: lymphocyte-to-C-reactive protein ratio, CRP: C-reactive protein, ALC: absolute lymphocyte count, s.c: superficial, HR: hazard ratio, 95% CI: 95% confidential interval, MFS: myxofibrosarcoma, UPS: Undifferentiated pleomorphic sarcoma, Cx: perioperative chemotherapy.

**Table 4:** Univariate and multivariate event-free survival analyses.

		Univariate Analysis			Multivariate Analysis		
Variables	Unit	HR	95% CI	*p* Value	HR	95% CI	*p* Value
Age	years	1.018	1.001–1.036	0.037	1.017	0.999–1.036	0.063
Sex	female	1					
	male	0.942	0.586–1.513	0.804			
Size	cm	1.108	1.064–1.154	<0.001	1.079	1.028–1.133	<0.001
depth	deep	1			0.81	0.379–1.73	0.586
	s.c	0.4751	0.235–0.959	0.038			
Grade	1	1					
	2	3.589	0.828–15.55	0.088	2.156	0.483–9.627	0.315
	3	6.171	1.498–25.42	0.012	3.385	0.79–14.5	0.101
Histology	MFS	1					
	UPS	1.442	0.673–3.087	0.347			
	Others	1.711	0.902–3.246	0.1			
Cx	No	1					
	Yes	1.635	0.987–2.709	0.0561			
LCR	High	1			1		
	Low	2.438	1.522–3.905	<0.001	1.462	0.849–2.52	0.171
CRP	mg/dL	1.187	1.123–1.255	<0.001	1.123	1.051–1.2	<0.001
ALC	/µL	0.999	0.999–1	0.676			

LCR: lymphocyte-to-C-reactive protein ratio, CRP: C-reactive protein, ALC: absolute lymphocyte count, s.c: superficial, HR: hazard ratio, 95% CI: 95% confidential interval, MFS: myxofibrosarcoma, UPS: Undifferentiated pleomorphic sarcoma, Cx: perioperative chemotherapy.

## Data Availability

No new data were created or analyzed in this study. Data sharing is not applicable to this article.
